# N‐methyl‐D‐aspartate receptor antibody production from germinal center reactions: Therapeutic implications

**DOI:** 10.1002/ana.25173

**Published:** 2018-03-25

**Authors:** Mateusz Makuch, Robert Wilson, Adam Al‐Diwani, James Varley, Anne‐Kathrin Kienzler, Jennifer Taylor, Antonio Berretta, Darren Fowler, Belinda Lennox, M. Isabel Leite, Patrick Waters, Sarosh R Irani

**Affiliations:** ^1^ Oxford Autoimmune Neurology Group, Nuffield Department of Clinical Neurosciences University of Oxford Oxford United Kingdom; ^2^ Department of Psychiatry University of Oxford Oxford United Kingdom; ^3^ Department of Neurology John Radcliffe Hospital, Oxford University Hospitals Oxford United Kingdom

## Abstract

**Introduction:**

N‐methyl‐D‐aspartate receptor (NMDAR) antibody encephalitis is mediated by immunoglobulin G (IgG) autoantibodies directed against the NR1 subunit of the NMDAR. Around 20% of patients have an underlying ovarian teratoma, and the condition responds to early immunotherapies and ovarian teratoma removal. However, despite clear therapeutic relevance, mechanisms of NR1‐IgG production and the contribution of germinal center B cells to NR1‐IgG levels are unknown.

**Methods:**

Clinical data and longitudinal paired serum NR1‐reactive IgM and IgG levels from 10 patients with NMDAR‐antibody encephalitis were determined. Peripheral blood mononuclear cells from these 10 patients, and two available ovarian teratomas, were stimulated with combinations of immune factors and tested for secretion of total IgG and NR1‐specific antibodies.

**Results:**

In addition to disease‐defining NR1‐IgG, serum NR1‐IgM was found in 6 of 10 patients. NR1‐IgM levels were typically highest around disease onset and detected for several months into the disease course. Moreover, circulating patient B cells were differentiated into CD19^+^CD27^++^CD38^++^ antibody‐secreting cells in vitro and, from 90% of patients, secreted NR1‐IgM and NR1‐IgG. Secreted levels of NR1‐IgG correlated with serum NR1‐IgG (*p* < 0.0001), and this was observed across the varying disease durations, suggestive of an ongoing process. Furthermore, ovarian teratoma tissue contained infiltrating lymphocytes which produced NR1‐IgG in culture.

**Interpretation:**

Serum NR1‐IgM and NR1‐IgG, alongside the consistent production of NR1‐IgG from circulating B cells and from ovarian teratomas suggest that ongoing germinal center reactions may account for the peripheral cell populations which secrete NR1‐IgG. Cells participating in germinal center reactions might be a therapeutic target for the treatment of NMDAR‐antibody encephalitis. Ann Neurol 2018;83:553–561

Immunoglobulin G (IgG) autoantibodies to the NR1 subunit of the N‐methyl‐D‐aspartate receptor (NMDAR) cause a severe and diffuse encephalitis characterized by psychosis, amnesia, and a complex movement disorder.^1^, ^2^ The autoantibodies are typically present in serum and cerebrospinal fluid (CSF) at disease onset and persist for several years despite various immunotherapies. Around 20% of patients have an associated ovarian teratoma which expresses the NR1 subunit of the NMDAR,^3^, ^4^ making this a likely site of primary immunization. This finding, together with the higher concentrations of NR1‐IgG in serum than CSF,^2^, ^5^ suggest that the autoantibodies are initially generated in the periphery. Indeed, especially given the recent description of NR1‐specific B cells and antibody‐secreting cells (ASCs) in the CSF, the consistent observation of NR1‐IgG intrathecal synthesis is most likely secondary to the cells, and some autoantibodies, crossing the blood–brain barrier after the primary systemic immunization.^1^, ^2^, ^6^, ^7^

Overall, around 50% of patients with NMDAR‐antibody encephalitis fail to respond to first‐line therapies with corticosteroids, intravenous immunoglobulin (IVIG), and/or plasma exchange. Of these refractory cases, 50% respond to second‐line therapies including anti‐CD20 monoclonal antibodies (eg, rituximab) or cyclophosphamide.^8^ Anti‐CD20 antibodies cause B‐cell depletion, and cyclophosphamide inhibits lymphocyte proliferation. Yet, given that ASCs express little CD20 and few proliferate, it remains unclear how these drugs function to ameliorate features of NMDAR‐antibody encephalitis. Moreover, recent small series of patients with NMDAR‐antibody encephalitis describe some success with the proteasome inhibitor, bortezomib, a plasma cell–directed therapy.^9^ Despite increasingly widespread clinical use of these varied empirical interventions, no study has examined the immunological basis of NMDAR‐antibody production.

Traditional concepts in immunology outline two largely competing mechanisms of medium‐ to long‐term antibody production.^10^, ^11^ The first hypothesizes that, throughout the disease, ongoing germinal center reactions generate NR1‐specific B cells. These reactions cyclically differentiate B cells into ASCs, which initially secrete NR1‐specific IgM and subsequently IgG. Thereafter, several of these B cells and ASCs spillover into the blood, and some may reach the central nervous system (CNS). The alternative model predicts that a single temporally remote germinal center immunization may generate a pool of NR1‐specific B cells, and NR1‐specific IgM generation is limited to this initial immunization. Thereafter, some of the B cells differentiate into long‐lived plasma cells (LLPCs), some of which retain CD19, and survive to secrete NR1‐directed IgG in niches such as bone marrow, and perhaps the CNS, often for many years. In this second model, there is no further de novo generation of antigen‐specific LLPCs or precursor B cells, and this model may explain lifelong immunity to several infectious agents.^12^

Dissection of these dichotomous pathways of antibody secretion has potential clinical relevance. The former hypothesis predicts that attenuation of the germinal center response should reduce antibody levels and hence disease progression and relapses, whereas the second hypothesis suggests that CD19‐directed drugs or bortezomib, which delete some ASCs including many LLPCs, should be effective therapies for NMDAR‐antibody encephalitis. To clarify whether germinal center responses represented an important mechanism for NR1‐antibody generation, we examined relevant serological and cellular parameters from peripheral blood in patients with NMDAR‐antibody encephalitis.

## Patients and Methods

A prospectively assessed consecutive cohort of 10 patients were recruited with known CSF NR1‐IgG antibodies who met diagnostic guidelines for definite NMDAR‐antibody encephalitis.^13^ From these 10 patients, clinical details, including treatments (Table and Supplementary Table 1), peripheral blood mononuclear cells (PBMCs; sampled at 1–112 months after symptom onset [median, 16; mean, 25]), and longitudinal sera, were obtained. At time of PBMC sampling, no patient had received rituximab; 1 had received cyclophosphamide. Written informed consent was obtained (ethical approval: REC16/YH/0013 and 16/SC/0224).

Available longitudinal serum samples were assessed for NR1‐specific IgG and IgM antibodies by live HEK293T‐cell‐based assays and diluted to end point, as described previously.^2^ Anti‐human IgM Fc µ‐specific (A21216; Invitrogen, Carlsbad, CA) and IgG Fc γ‐specific (709‐585‐098; Jackson Labs, Bar Harbor, ME) Alexa Fluor 594–conjugated secondary antibodies were used for live cell‐based assays, and protein G sepharose beads (17‐0618‐01; GE, Little Chalfont, UK) and anti‐IgM‐conjugated agarose beads (19935; Sigma‐Aldrich, St. Louis, MO) were used to confirm subclass specificities. IgM and IgG binding to live hippocampal neurons, cultured as described previously,^14^ was also assessed. A commercial antibody directed against the extracellular domain of the NR1 subunit was used for colocalization experiments (#AGC‐001; Alomone, Jerusalem, Israel). Control sera (n = 116) were obtained from patients with aquaporin‐4 antibodies (n = 25), neuropsychiatric lupus (n = 24), myelin‐oligodendrocyte glycoprotein antibodies (n = 25), multiple sclerosis (n = 17), and from 25 healthy controls.

Ficoll density‐isolated patient (n = 10) and healthy control (n = 10) PBMCs (2 × 10^5^) were cultured in 96‐well plates. Each well contained 150 µl of RPMI (5% fetal calf serum plus penicillin‐streptomycin) with select combinations of cytokines, including interleukin (IL)‐2 (50ng/ml; PeproTech, Rocky Hill, NJ), IL‐1β (1ng/ml; PeproTech), IL‐21 (50ng/ml; PeproTech), IL‐6 (10ng/ml; R&D Systems, Minneapolis, MN), tumor necrosis factor α (TNFα; 1ng/ml; Peprotech), B‐cell activating factor (BAFF; 200ng/ml; R&D Systems), and other immune factors, including CD40‐ligand (in soluble form, 50 ng/ml; R&D Systems; and membrane‐bound form, CD40‐ligand‐expressing 3T3 cells after irradiation at 70 Gy) and R848 (a toll‐like receptor agonist; 2.5 μg/ml; Enzo Life Sciences, Farmingdale, NY).

After 7 days in culture, cells were labelled at 4 °C with antibodies for flow cytometry. Antibodies were directed against CD3 (clone UCHT1, Pacific Blue; BioLegend, San Diego, CA), CD14 (clone HCD14, Pacific Blue; BioLegend), CD19 (clone SJ25C1, ApC‐Cy7; BD Biosciences, San Jose, CA), CD27 (clone O323; BV605, BioLegend), CD20 (clone 2H7, FITC; BD Biosciences), IgD (clone IA6‐2, PE‐CF594; BD Biosciences), IgM (clone G20‐127, APC; BD Biosciences), IgG (clone G18‐145, Alexa Fluor 700; BD Biosciences), CD38 (clone HB7, PE‐Cy7; BD Biosciences), and CD138 (clone B‐B4, PE; Miltenyi Biotec, Somerville, MA). A BD LSRII flow cytometer and FlowJo (v10.1r5; FlowJo, LLC, Ashland, OR) software was used for analysis.

In parallel experiments, after 14 days in culture, nonsaturating quantities of 5‐10 μl of cell culture supernatants were tested for total IgG levels (IgG ELISA set; Bethyl Laboratories, Montgomery, TX). Another 50 μl of culture supernatant was tested by live cell‐based assay and, if positive, end‐point diluted to quantify NR1‐IgG and IgM.

From 2 patients, ovarian teratoma explants or intratumoral lymphocytes were cultured under select conditions described above for PBMC experiments. In addition, 5 µm of formaldehyde‐fixed teratoma sections underwent automated staining using various commercial antibodies (CD3, Leica LN10; CD20, Dako [Carpinteria, CA] L26; CD138, Leica Microsystems [Wetzlar, Germany] MI15; CD38, Abcam [Cambridge, MA] ab140799; CD27, Abcam ab70103; and NR1, Thermo Fisher Scientific [Waltham, MA] 1H13L3) and developed with 3,3'‐diaminobenzidine, as per the manufacturer's instructions (Leica Biosystems).

### Statistical Analysis

GraphPad Prism (version 7; GraphPad Software Inc., La Jolla, CA) was used for statistical analyses and data presentation.

## Results

**Figure 1:**
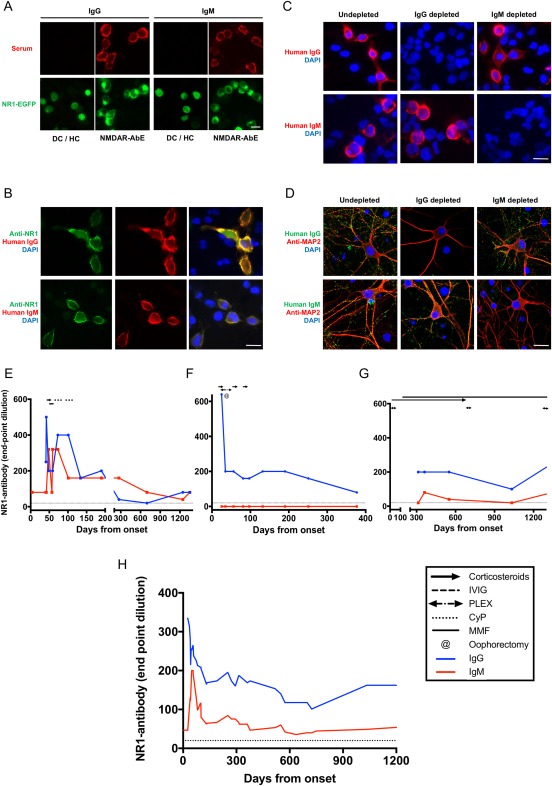
NR1‐subunit–specific IgG and IgM antibodies. (A) Live cell‐based assays expressing surface NR1/enhanced green fluorescent protein (EGFP) as a fusion protein (green), as described previously.^2^ IgG and IgM antibodies were detected among patients with NMDAR‐antibody encephalitis (NMDAR‐AbE; 1:20 serum dilution, red). One of 116 of the disease controls (DC) and healthy controls (HC) showed NR1‐IgM antibodies. No DC/HC showed NR1‐IgG. Scale bar, 10 μm. (B) Both NR1‐reactive IgM and IgG (red) colocalized well with an anti‐NR1 commercial antibody (green). Scale bar, 10 μm. (C) NR1‐IgM reactivity was specifically depleted with anti‐IgM precipitation, whereas protein G depletion of IgG abrogated all NR1‐IgG binding in live NR1‐expressing HEK cells. Scale bar, 10 μm. (D) Similarly, these reciprocal serum depletions confirmed specificity of both high‐titer NR1‐specific IgM and IgG antibodies which bound the surface of live hippocampal neurons (microtubule associated protein 2, MAP2, in red). Scale bar, 20 μm. Depletion was > 99%. (E–G) are representative figures of three observed trends in individual patients, and dotted line at serum dilution of 1:20 indicates cutoff for positivity. IgG is represented in blue and IgM in red, throughout. (E) Two of 10 patients showed parallel reductions of both NR1‐IgM and NR1‐IgG. (F) Four of 10 patients had NR1‐IgG without NR1‐IgM. (G) Four of 10 showed low‐level sustained fluctuations in NR1‐IgM levels. (H) Overall, the mean serum NR1‐IgM and IgGs of the 6 patients with NR1‐IgM were compared by end‐point dilutions. Values of neighboring points were used to smooth data (GraphPad Prism v7; 0th order polynomial). CyP = cyclophosphamide; DAPI = 4',6‐diamidino‐2‐phenylindole; Ig = immunoglobulin; IVIG = intravenous immunoglobulin; MMF = mycophenolate mofetil; NMDAR = N‐methyl‐D‐aspartate receptor; PLEX = plasma exchange.

All 10 patients (mean age, 29 years; range, 16–71; all female) had serum NR1‐IgG antibodies. In addition, NR1‐specific IgM antibodies were detected in the first available serum samples from 6 of 10 (60%) patients (Fig 1A; Table). NR1‐IgG and NR1‐IgM specificities were confirmed by consistent colocalization with surface NR1‐EGFP (enhanced green fluorescent protein) expression (Fig 1A) and with an anti‐NR1 commercial antibody (Fig 1B). Furthermore, in both live NR1‐transfected cells and in live hippocampal neuronal cultures, complete depletion of either IgG (with protein G) or IgM (with anti‐IgM‐coated beads) abrogated NR1‐IgG or NR1‐IgM binding, respectively (Fig 1C,D). In addition, none of the NR1‐reactive sera bound to leucine‐rich glioma inactivated‐1 or aquaporin‐4‐transfected HEK cells (data not shown), and NR1‐IgM were present in only 1 of 116 healthy and disease control sera (Fig 1A; *p* < 0.0001, Fisher's exact *t* test, 1 patient with aquaporin‐4 antibodies).

Longitudinally, in 4 patients, serum NR1‐IgM was detected at low levels (1:20–1:100 end‐point dilutions), in 2 patients they closely mirrored NR1‐IgG levels (up to 1:640 end‐point dilutions) and in 4 patients NR1‐IgM were absent throughout the disease course (representative examples in Fig 1E–G). Overall, when present, the NR1‐IgM levels were highest around disease onset and were detected at 50% of their peak levels after approximately 6 months (Fig 1H). This duration is far longer than the 5‐day half‐life of IgM,^15^ and strongly suggested ongoing production rather than persistence of NR1‐IgM. This active production implicated antigen‐specific germinal center responses continued for several months into the course of NMDAR‐antibody encephalitis.

**Figure 2:**
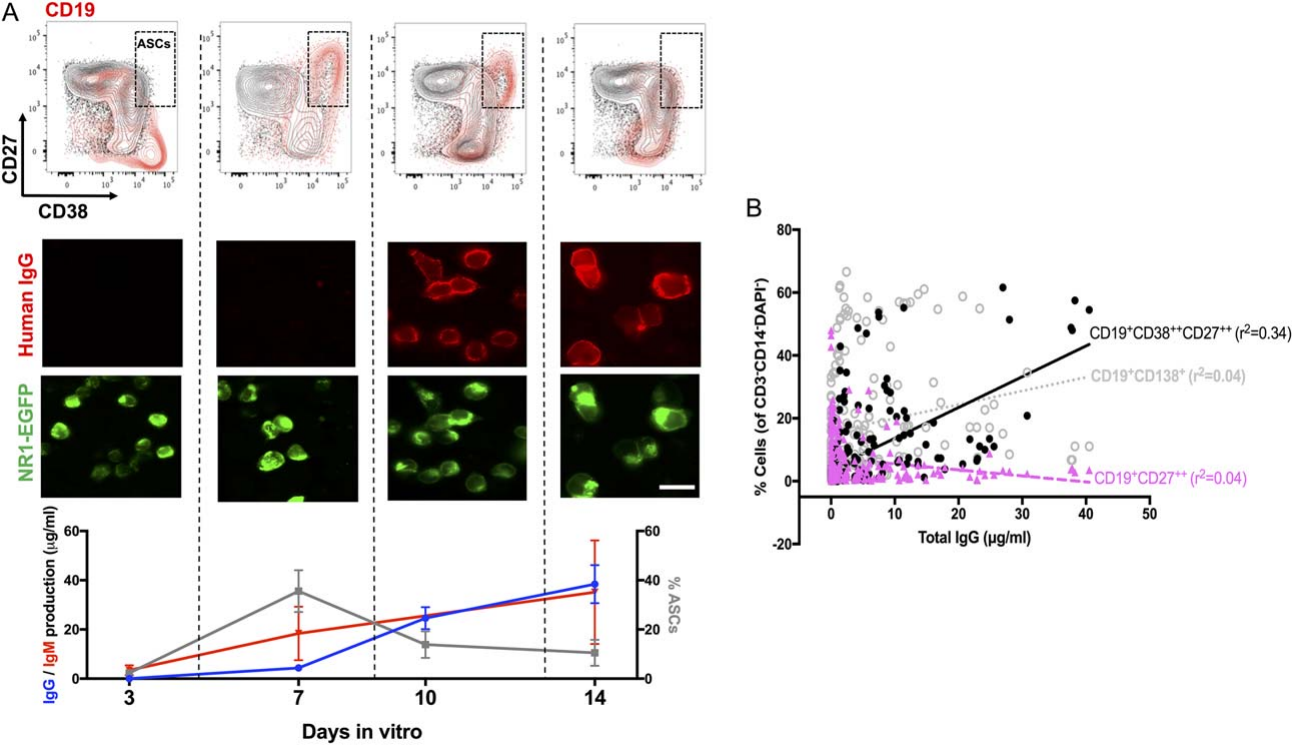
In vitro production of NR1‐directed antibodies from PBMC cultures. (A) Antibody‐secreting cells (ASCs) reach a peak at day 7. (B) This CD19^+^CD27^++^CD38^++^ population of ASCs correlated best with the total IgG produced in culture. From (A), total IgG and IgM production (µg/ml) plateaued at around day 14, and NR1 antibodies could be detected in some culture supernatants. Time course is plotted for experiments from 5 patients using soluble CD40‐ligand, interleukin‐2, and the toll‐like receptor 7/8 agonist, R848 (mean and SD shown). Scale bar, 10 μm. EGFP = enhanced green fluorescent protein; Ig = immunoglobulin; PBMC = peripheral blood mononuclear cell.

Next, to understand whether the germinal centers were also actively generating NR1‐specific B cells, circulating B cells were differentiated into ASCs to examine NR1‐antibody secretion. From unfractionated PBMC cultures, in vitro generation of human ASCs was especially efficient under conditions that included CD40‐ligand, IL‐2, and toll‐like receptor stimulation (R848), consistent with T‐cell help and previous reports (Fig 2A).^10^ These conditions, and less so those with IL‐21, generated a substantial population of CD19^+^ CD27^++^CD38^++^ ASCs at day 7 in vitro (Fig 2A), and 25% of the CD19^+^CD27^++^CD38^++^ cells (range, 15–38) expressed CD138, a known ASC‐subset marker. The peak CD19^+^CD27^++^CD38^++^ population correlated well with the total IgG produced in vitro (Fig 2A,B; r^2^ = 0.34; *p* < 0.0001). Indeed, by day 14, total IgM and IgG secretion had plateaued and, intriguingly, NR1‐IgM and ‐IgG became detectable in some supernatants (Fig 2A).

**Figure 3:**
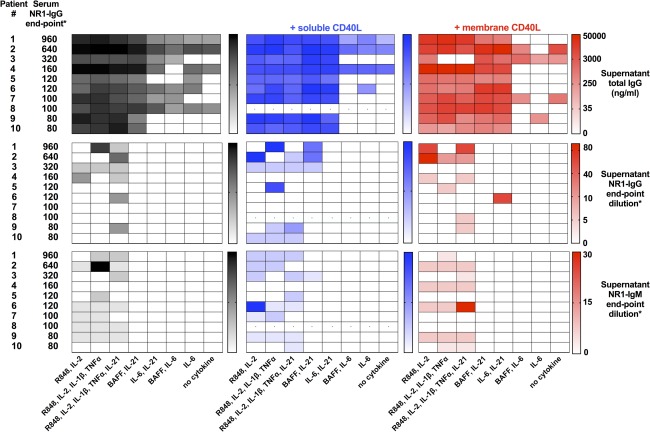
NR1‐specific IgG and total IgG production in vitro. Production of total IgG (upper heatmaps; ng/ml), NR1‐specific IgG (middle heatmaps), and NR1‐IgM (lower heatmaps) from 10 patients with NMDAR antibody encephalitis. Patients (numbers corresponding to the Table) ordered in rows by descending serum NR1‐IgG levels at time of PBMC sampling. *Serum NR1‐IgG and supernatant NR1‐IgG and ‐IgM levels were determined by live cell‐based assay end‐point dilutions. Culture conditions are listed below each column and varied in use of CD40‐ligand (nil in black; soluble CD40‐ligand in blue; membrane CD40‐ligand in red). Those with CD40‐ligand, interleukin‐2, and R848 most frequently generated NR1‐antibodies. Heatmaps show gamma‐transformed data for visualization, and absolute levels are shown in the bars. Limited cell numbers meant 1 patient had unavailable data in soluble CD40‐ligand conditions (dots). BAFF = B‐cell activating factor; CD40L = CD40‐ligand; Ig = immunoglobulin; IL = interleukin; NMDAR = N‐methyl‐D‐aspartate receptor; PBMC = peripheral blood mononuclear cell; TNF‐α = tumor necrosis factor alpha.

Overall, as shown in Figure 3, exposure of the 10 patient PBMCs to eight varying experimental conditions revealed NR1‐IgG in one or more culture supernatants from 9 of 10 patients (90%). The NR1‐IgG were unlikely to have arisen from pre‐existing circulating ASCs derived ex vivo, given that they were not generated under conditions known to maintain ASCs in culture (IL‐6 ± BAFF).^16^ By contrast, under conditions known to proliferate circulating B cells (including IL‐2 and R848),^10^ NR1‐IgG was consistently detected in culture supernatants both with and without CD40‐ligand (Fig 3). NR1‐IgM was also observed in several wells, typically at lower levels. Therefore, both NR1‐specific IgG and IgM could be generated from peripheral B‐cell cultures, and neither were detected in culture supernatants from 10 age‐ and sex‐matched healthy controls.

Because NR1‐IgG has proven pathological relevance in NMDAR‐antibody encephalitis,^2^, ^8^, ^13^ next we explored factors related to its production. The total amount of NR1‐IgG generated in vitro for an individual patient correlated with their serum NR1‐IgG levels (r^2^ = 0.88; *p* < 0.0001; Supplementary Fig 1), and not with parameters including total IgG production in vitro (r^2^ = 0.38; *p* = 0.08), time since disease onset (r^2^ < 0.01; *p* = 0.83), time since immunotherapy initiation (r^2^ = 0.10; *p* = 0.40), or types of immunotherapy (data not shown). Indeed, this NR1‐IgG production was observed at a variety of times from disease onset (see Table).

**Figure 4:**
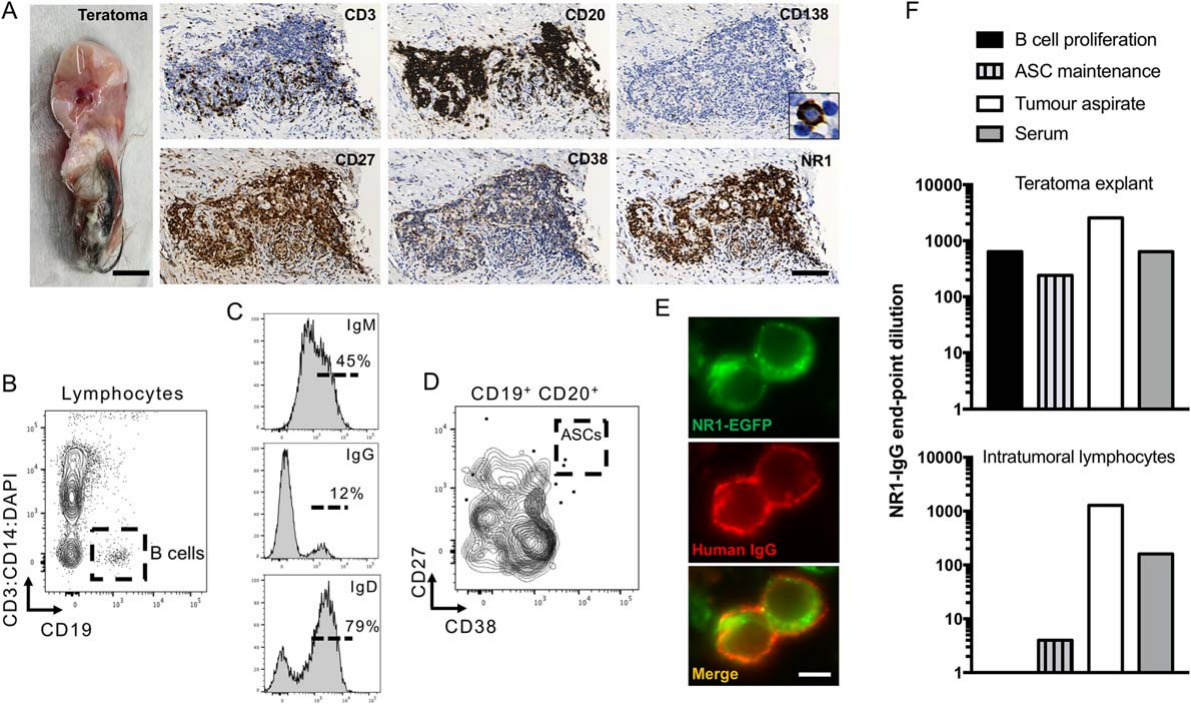
NR1‐IgG antibody generation from ovarian teratomas. (A) An ovarian teratoma from patient number 2 (scale bar, 0.2cm), showed dense B cell (CD20) infiltration, with more sparse T cells (CD3) and few plasma cells (CD138, inset × 80 magnification). T and B cells express CD27, whereas CD38 is more restricted to the B cell regions. The NR1 subunit is densely expressed throughout this region. Scale bar, 100 μm. (B) Cystic aspirate from another teratoma (a 28‐year‐old female recruited after patients 1–10) was studied by flow cytometry and found to contain CD19^+^ B cells, which (C) expressed surface IgM, IgG, and IgD. (D) In addition, some CD19^+^ cells were CD27^++^CD38^++^ antibody‐secreting cells (ASCs). (E,F) Culture of these cells with an ASC‐maintenance condition (interleukin‐6), but not B cell proliferative condition (R848, CD40‐ligand, and interleukin‐2), resulted in NR1‐IgG generation (E,F, lower panel). Scale bar, 5 μm. NR1‐IgG was also detected from supernatants of the teratoma explants of patient 2 (F, upper panel) after incubation with B cell proliferative conditions or interleukin‐6. In addition, NR1‐IgG was detected in aspirates directly from the teratoma at higher levels than in the serum from these 2 patients (F). DAPI = 4',6‐diamidino‐2‐phenylindole; EGFP = enhanced green fluorescent protein; Ig = immunoglobulin.

Finally, the capacity for NR1‐antibody generation from two ovarian teratomas was studied (Fig 4). The teratomas contained germinal center–like structures with numerous B cells (CD20), clusters of T cells (CD3), and scattered plasma cells (CD138; Fig 4A). The T cells and B cells both expressed CD27; CD38 was prominent in the B cell regions, and the NR1 subunit was densely expressed throughout. More detailed B cell analysis from the aspirate of a cystic teratoma confirmed presence of IgM, IgG, and IgD expressing CD19^+^ cells, in addition to less frequent CD27^++^CD38^++^ ASCs (Fig 4B–D). In culture, these aspirated cells and the teratoma explants both produced IgG (1–3 µg/ml) and IgM (1–4 µg/ml) and, more importantly, NR1‐IgG (Fig 4E,F). Overall, NR1‐IgG was produced both under conditions which favored maintenance of resident ASCs (IL‐6) and B‐cell proliferation (R848, soluble CD40‐ligand, and IL‐2; Fig 4F). These observations, together with the higher NR1‐IgG levels observed in tumor aspirates than patient serum (Fig 4F), confirm intratumoral synthesis of NR1‐IgG, likely from a combination of resident B cells and ASCs.

## Discussion

These experiments show that, for several years through the course of NMDAR‐antibody encephalitis, NR1‐specific IgM can be detected in patient sera, together with NR1‐specific B cells both in the circulation and in the teratoma tissue. Furthermore, under the experimental conditions presented, the circulating B cells can produce NR1‐IgG at levels proportional to serum NR1‐IgG. These collective findings suggest that persistent germinal center activity may be responsible for the ongoing production of NR1‐IgG and argue against a dominant contribution from LLPCs to circulating NR1‐IgG.^9^, ^17^, ^18^ Based on these data, future immunotherapies might aim to interfere with the active germinal center responses in NMDAR‐antibody encephalitis to terminate ongoing antibody production.

This study has several other important clinical implications. First, the in vivo differentiation of these NR1‐specific B cells into short‐lived ASCs may mediate the observed correlation between the in vitro NR1‐IgG generation and serum NR1‐IgG levels. If so, rituximab might be partially effective in reducing NR1‐specific B cells.^8^ However, the efficacy of rituximab may be limited by the known relative insensitivity of lymph node–resident B cells, as observed in 1 postmortem case of NMDAR‐antibody encephalitis.^18^, ^19^ Alternatively, it remains possible that the bone marrow–niched LLPCs produce NR1‐IgG at levels proportional to the circulating B cell‐derived ASCs, and this possibility can only robustly be addressed with lymphocyte cultures directly from bone marrow samples. However, two pools of preformed active antigen‐specific ASCs—one in bone marrow and another in circulation—would be inconsistent with traditional models of antibody secretion,^11^ and would not account for the duration of NR1‐IgM production which we observed. To date, studies that found NR1‐IgM have not related their presence to ongoing de novo production from recent germinal center reactions.^20^, ^21^ However, in our study, NR1‐IgM rates were surprisingly high in NMDAR‐antibody encephalitis patients and low in disease controls.^22^ The higher detection rate in the target population may be explained by use of live cell‐based assays, which are known to enhance sensitivity of NR1‐IgG detection.^23^ Furthermore, perhaps exclusion of intracellular epitope binding explains the low rate observed in disease controls. Also, few PBMCs were sampled in the acute phase, and none preceding immunotherapy: these aspects require further study in larger cohorts. Nevertheless, this human cell culture model appears translatable to ongoing similar studies in patients with neuromyelitis optica^24^ and faciobrachial dystonic seizures^25^ (Makuch, Waters and Irani, unpublished), suggesting the presence of circulating antigen‐specific B cells in patients with many autoantibody‐mediated conditions.

Second, it is of potential etiological interest that NR1‐specific B cells were efficiently expanded in vitro by toll‐like receptor agonism because this is an established property of herpes simplex virus (HSV),^26^ and HSV is both a known trigger of classical NMDAR‐antibody encephalitis,^27^, ^28^ and of NR1‐IgG and NR1‐IgM generation in patients without typical clinical features of NMDAR‐antibody encephalitis.^21^, ^29^ However, this hypothesis would necessitate preformed NR1‐reactive cells. Also, toll like receptor activation may expand T cells within PBMC cultures. Alternatively, given that HSV often causes de novo generation of NMDAR‐antibodies, it may expose CNS antigens, including the NMDAR, which then preferentially initiate an immune reaction in the draining cervical lymph nodes.^30^ As yet unknown immunogens may also operate through this mechanism in NMDAR‐antibody encephalitis.

Third, together with ongoing NR1‐IgM production, the presence of circulating B cells with the capacity to produce NR1‐antibodies may suggest that lymph nodes are actively producing NR1‐reactive B cells. This effect may be through antigen‐dependent B cell generation or through polyclonal bystander activation of preformed memory B cells. By analogy, the ovarian teratomas express the NR1 autoantigen and neurons with abnormal morphology, which may break immune tolerance.^3^, ^4^ In addition, we and others have observed intra‐teratoma lymphocyte‐rich structures with dense T and B cell infiltrations,^31^ and our data show that these tumor‐resident B cells also have the capacity to generate NR1‐specific antibodies. Therefore, the ovarian teratoma contains foci highly reminiscent of germinal center structures, and these collective observations provide a scientific basis to explain why early teratoma removal improves patient outcomes.^1^, ^2^, ^8^ However, NR1‐specific cells may exit this putative site of immunization and seed other lymphoid organs, explaining ongoing antibody production after teratoma excision. Because both lymph nodes and teratomas contain germinal center–like structures, it is intriguing that somatic hypermutation was limited in many NR1‐specific B cells isolated from CSF.^6^ Given that NR1‐reactive IgM and IgA are frequently detected in many disease populations,^20^ and CD40‐ligand had a limited influence on NR1‐IgG production in our study, it may be that naïve autoreactive B cells with few immunoglobulin mutations are sufficient for pathogenic NR1‐IgG production. Nevertheless, it should be noted that some patients had undetectable NR1‐IgM levels, and teratomas are observed in the minority of patients, and hence it remains possible that germinal center reactions may not be active in all patients.

Finally, the presence of active germinal centers in the periphery, together with the higher absolute levels of NR1‐IgG in serum than CSF and the disease association with ovarian teratomas, all strongly support the initial peripheral generation of NR1 antibodies.^2^, ^5^ How the peripherally generated NR1‐specific cells cross the blood–brain barrier and are retained to produce relative intrathecal synthesis of NR1‐IgG, upon normalization for total IgG levels, is a separate question, and it may be that their activation in the periphery is sufficient to induce ligands which facilitate transmigration into the CNS.

In summary, this study demonstrates: NR1‐IgG and ‐IgM production from circulating PBMC cultures, teratoma‐based germinal center–like structures which can generate NR1‐IgG, and the detection of serum NR1‐IgM for long durations after disease onset. Taken together, these data suggest that germinal centers have the capacity to generate NMDAR‐antibodies. Therapeutic interventions in humans with NMDAR‐antibody encephalitis are required to further examine this hypothesis.

## Author Contributions

S.R.I., M.M., R.W., and A.A.D. contributed to study concept and design, data acquisition and analysis, and drafting the manuscript and figures. J.V., A.K.K., J.T., A.B., D.F., B.L., M.I.L., and P.W. contributed to data acquisition and analysis.

## Potential Conflicts of Interest

S.R.I. and P.W. are co‐applicants and receive royalties on patent application WO/2010/046716 entitled “Neurological Autoimmune Disorders.” The patent has been licensed to Euroimmun AG for the development of assays for LGI1 and other VGKC‐complex antibodies.

**Table 1:** Clinical and Serological Features of Patients With NMDAR Antibody Encephalitis at Time of PBMC Sampling

Patient No.	Age, y	Serum NR1‐IgG End‐Point Dilution	Serum NR1‐IgM End‐Point Dilution	Duration Since Disease Onset	Treatments	Duration of Immunotherapy
1	56	960	0	1,237	Pred, MMF	1,192
2	16	640	0	81	IVMP	57
3	22	320	320	59	IVIG	17
4	25	160	160	3,377	Nil	NA
5	17	120	160	348	Pred	273
6	19	120	20	295	Pred	245
7	26	100	40	797	Pred	736
8	22	100	0	635	Pred	148
9	26	80	0^a^	40	Pred, IVIG	18
10	71	80	0	767	IVMP, Pred	645

The clinical features of 10 female patients whose peripheral blood mononuclear cells (PBMC) were utilized in experiments. Treatments, durations, and NR1‐IgG and NR1‐IgM end‐point dilutions were evaluated at time of sampling.

a Patient 9 had NR1‐IgM detected from their first serum sample (1:20 end‐point dilution). All durations in days.

Ig = immunoglobulin; IVIG = intravenous immunoglobulin; IVMP = intravenous methylprednisolone; MMF = mycophenolate mofetil; mRS = modified Rankin Score; Pred = oral prednisolone.

## Supporting information

Additional supporting information can be found in the online version of this article.

Supporting Information
