# Supplementary material for: N‐methyl‐D‐aspartate receptor antibody production from germinal center reactions: Therapeutic implications
Source: Ann Neurol. 2018 Mar 25;83(3):553–61. doi: 10.1002/ana.25173 (PMC5925521; doi:10.1002/ana.25173)
Supplement: Supplementary file 1 — Supporting Information [file ANA-83-553-s001.docx]

| **Patient number** | **Ethnicity** | **Time since last relapse** | **Peak mRS** | **mRS at sampling** | **Treatments prior to PBMC sampling** | **Treatments after PBMC sampling** |
| --- | --- | --- | --- | --- | --- | --- |
| 1 | Caucasian | 248 | 5 | 2 | IVMP, Pred, PLEX, IVIG, MMF | Pred |
| 2 | Asian | NA | 5 | 2 | IVMP, PLEX, Oophorectomy | Nil |
| 3 | Asian | NA | 5 | 5 | IVMP, IVIG | CyP |
| 4 | Caucasian | 2312 | 5 | 0 | IVMP, Pred, IVIG, PLEX, AZA | Nil |
| 5 | Caucasian | NA | 4 | 0 | IVMP, Pred, IVIG, CyP | Nil |
| 6 | Caribbean | NA | 4 | 2 | IVMP, Pred, PLEX | Nil |
| 7 | Caucasian | NA | 5 | 1 | IVMP, Pred, PLEX | IVMP, PLEX, CyP |
| 8 | Caucasian | NA | 3 | 2 | IVMP, PLEX | Pred |
| 9 | Caucasian | NA | 5 | 4 | IVMP, Pred, IVIG | PLEX, CyP |
| 10 | Asian | 37 | 5 | 4 | IVMP, Pred, PLEX | CyP, MMF |

**Supplementary Table 1. Clinical and treatment details of patients whose peripheral blood mononuclear cells (PBMCs) were used in experiments.** AZA = azathioprine; CyP = cyclophosphamide; IVIG = intravenous immunoglobulins; IVMP = intravenous methylprednisolone; MMF = mycophenolate mofetil; mRS = modified Rankin Score; PLEX = plasma exchange; Pred = oral prednisolone

**Supplementary Figure 1. NR1-IgG production in culture.** The total amount of NR1-IgG production in culture per patient associated most closely with patient serum NR1-IgG levels determined by end-point dilutions (r^2^=0.88, p<0.0001), and not with total IgG production *in vitro (*r^2^=0.38, p=0.08*)*, time since disease onset (r^2^<0.01, p=0.83) or time since immunotherapy initiation (r^2^=0.10, p=0.40).
